# Gut-brain axis and neurodegeneration: mechanisms and therapeutic potentials

**DOI:** 10.3389/fnins.2024.1481390

**Published:** 2024-10-23

**Authors:** Kelly Jimin Park, Yao Gao

**Affiliations:** ^1^Pomfret School, Pomfret, CT, United States; ^2^Vascular Biology Program, Boston Children’s Hospital, Boston, MA, United States; ^3^Department of Surgery, Harvard Medical School, Boston, MA, United States

**Keywords:** gut-brain axis, neurodegeneration, neuroinflammation, gut microbiota, metabolites, mechanisms, therapeutic potentials

## Abstract

This paper reviews the effects of gut microbiota in regulating neurodegenerative diseases through controlling gut-brain axis. Specific microbial populations and their metabolites (short-chain fatty acids and tryptophan derivatives) regulate neuroinflammation, neurogenesis and neural barrier integrity. We then discuss ways by which these insights lead to possible interventions - probiotics, prebiotics, dietary modification, and fecal microbiota transplantation (FMT). We also describe what epidemiological and clinical studies have related certain microbiota profiles with the courses of neurodegenerative diseases and how these impact the establishment of microbiome-based diagnostics and individualized treatment options. We aim to guide microbial ecology research on this key link to neurodegenerative disorders and also to highlight collaborative approaches to manage neurological health by targeting microbiome-related factors.

## Introduction

**FIGURE 1:**
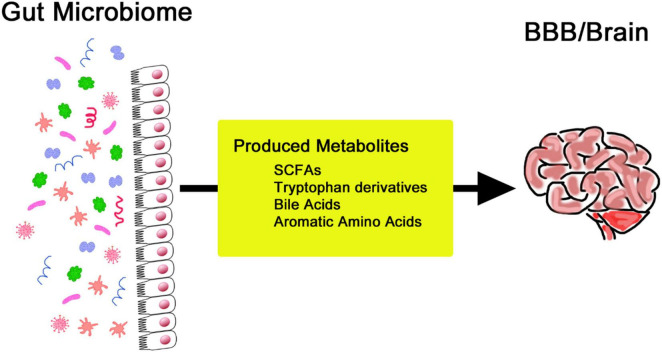
Simplified illustration of the bidirectional communication network linking the intestinal microbiome and the central nervous system.

The emerging field of microbiome research is changing our understanding of human biology and health and its interaction with the environment (Lu et al., 2014; Manolis et al., 2022). Over the past decade, research has revealed many new aspects of our role as a host for trillions of microbes and many physiological processes they serve (Kurilshikov et al., 2017; Bellono et al., 2017). The gut-brain axis is relatively less explored but can be used to directly impact effect on clinical research; it is defined as the two-way communication network that connects intestinal microbiome with the central nervous system (CNS) (O’Mahony et al., 2015; Han et al., 2018). This bidirectional communication involves multiple layers of communication pathways, encompassing the immune, nervous, and circulatory systems (Figure 1). The gut-brain axis is recognized as one of the major pathways by which properties and health status of the microbiome can influence not just brain health but also various types of diseases across all other systems in the body (Di Meo et al., 2018; Bonaz and Bernstein, 2013). Alzheimer’s, Parkinson’s, and Huntington’s diseases are some examples of neurodegenerative disorders that are characterized by progressive neuronal loss and dysfunction over time leading to decreased cognition and motor functions (Dugger and Dickson, 2017; Lamptey et al., 2022). Despite extensive research that has been conducted so far to understand and treat such neurodegenerative diseases, their pathogenesis is not fully understood. As a result, most current therapies focus to manage symptoms rather than addressing the underlying causes (Bloom, 2014; Jellinger, 2010). Gut microbiomes play key roles in neurological disorders, mainly through regulating inflammation, communication, and metabolism with the CNS (Kaur et al., 2019; Begum et al., 2022).

While the gut-brain connection is not new (Bonaz and Bernstein, 2013; Dinan and Cryan, 2017), advances in high-throughput sequencing and bioinformatics now allow deeper analysis of complex microbial communities (Lu et al., 2014; Kurilshikov et al., 2017; Gohl et al., 2016; Bell et al., 2019). There are emerging links between diet, microbiota composition and neuro-health that interact in complex ways, and they show promise for personalized microbiome-based interventions (Han et al., 2018; Di Meo et al., 2018; Gubert et al., 2020; Cryan et al., 2019). In this review, recent findings on how the gut microbiome could influence neurodegenerative disease will be covered. We examine epidemiological and clinical evidence that intestinal microbiota composition changes in relation to neurodegenerative processes before symptoms appear. We also summarize a number of therapeutic approaches for disease modification targeting gut microbiome (Gubert et al., 2020; Sampson et al., 2016; Toledo et al., 2022). From a multidisciplinary perspective, including microbiology, neuroscience, immunology, and clinical medicine, we discuss preventive and therapeutic strategies. These strategies target gut microbial communities to modulate the gut-brain axis that may be linked to neurodegeneration (O’Mahony et al., 2015; Bonaz and Bernstein, 2013; Gubert et al., 2020; Fang et al., 2020).

## Overview of the gut microbiome

New interest in the field of gut microbiome has increased in recent years thanks to initiatives like the Human Microbiome Project and the discovery of techniques such as FMT (Kho and Lal, 2018; Mohajeri et al., 2018). Such studies have greatly expanded current knowledge of how systems like the gut-brain axis intersect with unrelated endogenous processes of diseases or phenotypes. The standard bacterial ecosystem of the body is complex, and studies have demonstrated that numerous components can shape this microbial community, including diet, age, and environment (Lu et al., 2014). The gut microbiome works on complex carbohydrates and fibers. This process has direct impact on energy balance and metabolic health of the host, so it indirectly indicates participation from a metabolic pathway. The gut microbiota also plays a role in the development and maturation of the immune system. It modulates systemic immunity, enhances resistance to pathogens, and therefore is an important mechanism of protection from exogenous microbes. It also helps that the gut microbes produce essential vitamins and conjugate bile acids required to break down fat. This metabolic role of the gut microbiome is exemplified by the fermentation of dietary fibers into short-chain fatty acids (SCFAs), one type of host signaling molecule and energy source (Manolis et al., 2022).

**FIGURE 2:**
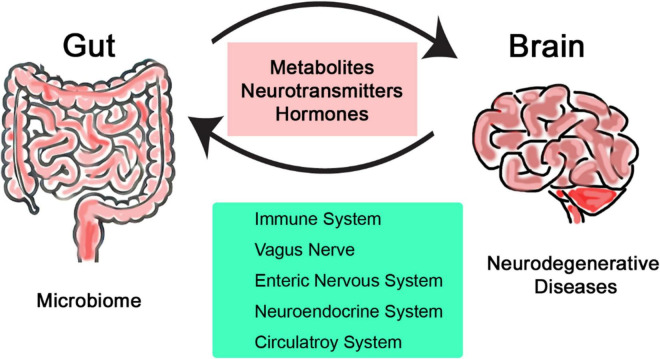
Production of various metabolites by the gut microbiome to regulate the blood-brain barrier and brain activities.

Substantial changes exist throughout the human lifespan, which are often ascribed to the changing needs of the body, which include bodily balance and protection from disease (Yatsunenko et al., 2012). The composition of one’s gut microbiome is easily expressed, with regional differences and cultural backgrounds providing variants of lifestyle practices, environmental exposures, and, most especially, dietary practices (Kurilshikov et al., 2017). Many other factors that further dictate the gut microbiota include medication use, level of stress, sleeping patterns, and physical activity. These factors cause changes in the microbial community, hence influencing its composition and functionality (Yatsunenko et al., 2012). The gut-brain axis is increasingly recognized as a channel through which the digestive system communicates with the CNS, a relationship linked with intricate neural interactions. In this context, the vagus nerve makes an essential contribution to connecting the gut and brain and, therefore, is crucial for understanding neural mechanisms behind mood disorders and paves the way for new therapies (Bellono et al., 2017; Han et al., 2018). Gut microbiota acts through hormonal signaling, a key mechanism in gut-brain communication. For example, gut microbiota-associated alterations in tryptophan metabolism decrease serotonin function in the brain and contribute to mood disorders accompanied by gastrointestinal dysfunction (O’Mahony et al., 2015). This kind of modulation can also extend the ability to perform cognitive and emotional processing. Signaling molecules like SCFAs play a critical role in brain-gut interactions (Bonaz and Bernstein, 2013). Artificial synthesis of these metabolites may be a new therapeutic target for neurodegenerative pathologies. SCFAs are produced by the fermentation of dietary fiber by gut bacteria (Figure 2). They significantly impact cognitive function and overall health (Di Meo et al., 2018). SCFAs influence neuroinflammation through receptor-mediated mechanisms and support the integrity of the blood-brain barrier (BBB) by promoting neurogenesis. These metabolites are also key modulators of neuroinflammation, BBB function, and cell growth. They are deeply interlinked and determine cognitive abilities and protect against various brain diseases (Wenzel et al., 2020; Liu et al., 2020; Fock and Parnova, 2023).

## Neurodegenerative disorders

Neurodegenerative diseases are mainly caused by progressive losses of neurons or other neurological cells within the CNS (Gao and Hong, 2008). The pathologies and phenotypes of these neurodegenerative disorders can vary considerably, and the causes and mechanisms also differ for most of these diseases. These cell losses make neurons especially prone to damage (Dugger and Dickson, 2017). The selective permeability of the BBB restricts the entry of external substances, complicating the treatment design. Most treatments need a polar component to facilitate transport in the bloodstream, but they must also avoid being too polar to cross the lipid bilayer of the BBB. Additionally, the regenerative capacity of the BBB is much slower compared to all other body systems so that it is highly vulnerable to injury and functional decline (Lamptey et al., 2022). Dementia, including Alzheimer’s disease, involves progressive cognitive decline and daily functional impairment. It is one of the most age-related causes of death in the United States, however, its potential causes and treatments still remain incompletely understood (Goyal et al., 2021). Current hypotheses proposes that Alzheimer’s pathology consists of extracellular amyloid plaques and intraneuronal neurofibrillary tangles made up of amyloid-β peptides and tau proteins. These toxic deposits interfere with synaptic functions critical for cognition and memory, and also trigger synapse loss through impairment of axon and dendrite maintenance or neuron death (Bloom, 2014). All these symptoms often overlap with normal aging-related phenotypes and very hard to detect at an early stage. Another example is Parkinson’s disease - characterized by motor symptoms such as slow movement, rigidity and balance problems that progressively complicate movement with neurodegeneration advancement. It is thought that dysfunction of dopaminergic neurons in various parts of the brain is the root of the disorder; thus, it is difficult to diagnose because of a wide range of symptomatology. Such degeneration of the neurons within the substantia nigra pars compacta is a common pathology in Parkinson’s. Consequently, dopamine signaling in the striatum is disrupted, which in turn disrupts motor cortical and basal ganglia circuits, and manifest as movement disorders. These disturbances can significantly affect quality of life and have wide societal and economic consequences (Mazzoni et al., 2012). Therapeutically, Parkinson’s and other neurodegenerative disorders are a major focus of research.

Exact causes or mechanisms of neurodegenerative disorders remains largely unknown. Both genetic and environmental factors may contribute to disease development. Diseases are also often classified according to genetic pathways and the essential proteins implicated in their pathology (Jellinger, 2010). A common feature of neurodegenerative diseases is harmful effects of misfolded proteins that result in aggregation and accumulation of proteins and plaques inside a cell that contribute to dysfunction and ultimately cell death. Because of these features, the core cellular mechanism underlying misfolded protein folding and aggregation remain consistent. Current studies have supported the view that protein aggregates can spread- misfolded protein aggregates- in a manner similar to infectious diseases (Soto and Pritzkow, 2018). These have multiple causes at both genetic and environmental levels. For example, a single mutation to the parkin gene can increase the risk of glycosylated alpha-synuclein that results in early-onset of Parkinson’s (Bertram and Tanzi, 2005). Likewise, neurotoxic metals and pesticides also contribute to Alzheimer’s disease through oxidative stress or inhibition of protein-degrading enzymes. Actual administration of many of these various compounds implicated in neurodegenerative diseases in animal models has demonstrated the triggering of oxidative stress. In addition, gene expression changes affected by diet and environment play an important role in causing the diseases (Chin-Chan et al., 2015).

Neurodegenerative diseases present with cognitive, motor and non-motor symptoms. The cognitive impairments of Alzheimer’s Disease are characterized by features of memory loss, difficulty in the use of language, and progressive mental deterioration (Wattmo et al., 2013). Four key characteristics of Parkinson’s disease, resting tremor, bradykinesia, rigidity and postural instability, result in movement abnormalities and gait disturbances (Jankovic, 2008). In addition to cognitive and motor symptoms, neurodegenerative diseases are associated with non-motor symptoms, mood disorders and sleep disturbances. These symptoms indicate cognitive decline and have significant consequences for quality of life (Claassen et al., 2010; Baquero and Martín, 2015). REM Sleep Behavior Disorder is a condition that is characterized by abnormal movements during sleep, accompanied by vocalizations (Rodriguez et al., 2017). Treatments include cholinesterase inhibitors, namely, donepezil, rivastigmine, and galantamine. These treatments reduce cognitive impairment by increasing acetylcholine in the brains of Alzheimer’s disease. For Parkinson’s disease, the most prescribed medication is Levodopa in combination with carbidopa. It raises dopamine levels and alleviates motor symptoms (White and Hawke, 2003). A combined approach to all different disease factors is considered a promising strategy to develop pharmaceutical solutions for such conditions (Van der Schyf, 2011). Future treatments focusing on neuronal bioenergetics may more likely offer success to treat such disorders (Onyango et al., 2021). Rehabilitative therapies like physical, occupational, and speech are helpful to manage these diseases, which maintains the existing patient capabilities and help make life somewhat better for those living with these conditions. Neurodegenerative diseases are difficult to address since they are likely due to multiple factors in nature and limited effectiveness of current therapies. Most of the treatments described above mainly provide symptomatic relief, but do not change the course of those conditions. For example, therapeutic measures to fight the effects of Alzheimer’s disease are likely to provide limited benefits only, as their effect wears off with time and complicates the clinical management of such patients (Kowal et al., 2019). In Parkinson’s disease, Levodopa provides symptomatic relief, but does not prevent the disease course whereas its beneficial effect decreases with progressing disease (Davie, 2008). What makes it so difficult to find a cure for these diseases is because of the inability of nerves to regenerate. However, there is a speck of hope arising from the advancement in stem cell research in recent years. The key to the optimal management of the neurocognitive disorders is early and accurate diagnosis. Advanced diagnostic tools, including positron emission tomography (PET) or Single photon emission computed tomography (SPECT) scans, offer improved disease managing strategies (Seibyl, 2023). Additional research into effective treatments is needed to better understand the underlying molecular bases and how therapeutic approaches may or may not be effective in such diseases (Lanza et al., 2018).

## Relating neurodegeneration to gut microbiome

The gut-brain microbiome forms a complex network through which communication between the CNS and the digestive system takes place in both directions (Kuwahara et al., 2020). Increased leakiness of the gut and blood-brain barriers, secretion in the brain of amyloids and lipopolysaccharides, and inflammatory responses by the gut microbiota may affect neurodegenerative diseases progression (Gubert et al., 2020; Jiang et al., 2017). A study found that glutamine and serotonin, representing gut microbiota and metabolites respectively, increased the risk for Alzheimer’s and Parkinson’s diseases. Such finding shows potential of gut microbiota and their metabolites for being targets in therapy and give an insight into the accompanying mechanisms of neurodegenerative diseases (Ning et al., 2022). Another study examined how selected diet and lifestyle conditions influence the regulation of neurodegenerative disorders by gut microbiome and promote brain resilience or changes through non-drug interventions to target gut microbiome, diet and lifestyle (Raval et al., 2020). These suggest that lifestyle intervention could be an emerging area of research to better manage neurodegenerative conditions (Raval et al., 2020).

Preliminary findings indicate that the gut microbiome influences neurodegeneration through neuroimmune functions in the context of aging. It regulates the so-called ‘gut-to-brain axis,’ which is an important communication pathway of the gastrointestinal tract with the nervous system through stress-related mitochondrial pathways (Köhler et al., 2016). Dietary habits influence neuronal functioning and brain health directly and through gut microbiome changes in composition and adult neurogenesis indirectly (Ribeiro et al., 2020). SCFAs drived from the gut microbiome lower histone acetylation and enhance cellular waste removal. It impacts neurodegeneration and exemplifies complex interactions between gut microbiota and brain health (Shoubridge et al., 2021). The gut microbiota further regulates the neural stem cells activity within these brain regions where new neurons are formed and affects neurogenesis and brain aging in regard to neurodevelopmental age-related and neurodegenerative disorders (Sarubbo et al., 2022). Such findings provide a foundation to understand relationship between gut microbiota and neurodegenerative diseases.

The process of the gut microbiota imbalance, known as dysbiosis, results in a disturbance in gut microbial community with neurodegenerative diseases at different stages and severity (Chidambaram et al., 2022). Specific patterns of gut microbiome dysbiosis have been reported, which have recently been found to be neurodegeneration driven, correlates through chronic inflammation, protein misfolding and autoimmune dysregulation (Chidambaram et al., 2022). For example, alteration of gut microbiomes in Parkinson’s disease has been related to intestinal inflammation and gastrointestinal symptoms. It has been reported that both are increased in the course of that disease (Elfil et al., 2020; Romano et al., 2021). Modulation of diseases by modifying the gut microbiome might thus provide a promising therapeutic target. Early dietary intervention to improve gut health may also be beneficial in treatments for subtly predisposed progressive neurodegenerative disorders, and needs to be considered as promising therapeutic options. Examples include the Mediterranean diet, probiotics, and curcumin, which have been associated with the reduction of the rate of cognitive decline and improvement in gut health of Alzheimer’s disease via modification of gut microbiota (Leblhuber et al., 2021). The gut-brain axis, especially its interaction with components such as the toll-like receptors, has to be fully understood to claim that alterations influence the beginning stages of neurodegenerative diseases like Parkinson’s (Caputi and Giron, 2018). Systems biology approaches also examine such epigenetic effects in the role of personalized anti-inflammatory diets that could use control of our microbiome to lead to the prevention of neurodegenerative disease (Rosario et al., 2020).

In all neurodegenerative diseases, genetic predispositions along with environmental factors like diet and toxin exposure are considered key modulators of the gut microbiome. These same elements specifically influence the cause and progression of neurodegenerative disorders through alterations in the gut microbiota and interactions with neurological pathways (Dinan and Cryan, 2017; Wang H. et al., 2021). Most literature identifies that diet has the most significant impact on shaping the gut microbiota, so a variety of diet patterns determines a specific kind of diversity and dysbiosis among microbiota (Frausto et al., 2021). The composition and function of gut microbiota, and by extension the brain and neurodegenerative processes, can be influenced by the effects of cumulative psychological or physical stressors (Gubert et al., 2020). Among such contributory causes of increased vulnerability that lead to suffering from this set of illnesses, are environmental exposures like air pollution and ecological neurotoxins (Murata et al., 2022). The gut microbiome might respond to these environmental stressors through changes in its composition and metabolic activity that could act as contributing causes or enhancers of neurodegenerative pathologies (Narasimhan et al., 2021).

## Microbial metabolites and brain health

Microbial products play a very important role in nutrient absorption, immune function, and brain health (Dinan and Cryan, 2017). Gut-derived tryptophan metabolites are associated with neurological disorders and emphasize the link between microbiota and brain health (Kaur et al., 2019). High levels of indoxyl sulfate and trimethylamine-N-oxide represent the complex interaction between the gut and the brain (Lai et al., 2021). Microbial metabolites in the gut-brain axis revealing mechanisms of neurodegenerative disorders offer new treatment options that highlight the importance of maintaining gut and brain health (Cryan et al., 2019). SCFAs, produced by gut microbiota from dietary fibers, are not only important for digestive health but also for maintaining brain function. Among all SCFAs, butyrate has been found to have a great influence on neurodegenerative diseases. One study showed that butyrate may reduce neuroinflammation in Alzheimer’s disease models (Sun et al., 2020). These acids are involved in pathways that affect lipid and cholesterol metabolism (Dinan and Cryan, 2017). SCFAs significantly impact brain health by adjusting the BBB, reducing neuroinflammation, and improving neurogenesis. Butyrate A histone deacetylase inhibitor regulates gene expression and thus provides neuroprotection. A preclinical study for example demonstrated that butyrate can decrease amyloid β in Alzheimer’s disease (Lei et al., 2016). Excessive SCFAs may interfere with the activity of microglia and provoke the misfolding of α-synuclein involved in Parkinson’s pathology (Wiefels et al., 2024). On the other hand, it may induce amyloid β formation in Alzheimer’s due to a change in the phenotype of microglia (Colombo et al., 2021). SCFAs have dual roles showing that gut microbiota and brain function have complicated interactions and take key positions in neurodegenerative disorders. They further emphasize the therapeutic potential to treat Alzheimer’s and Parkinson’s, effects associated with an impact on neuroinflammation and cell signaling (Majumdar et al., 2023).

The kynurenine pathway of tryptophan metabolism is involved in brain function and mood regulation. Tryptophan was degraded mainly via the kynurenine pathway and resulted in the production of active compounds influencing the brain (Tan et al., 2012). Outcomes of produced compounds include kynurenine, kynurenic acid, and quinolinic acid (Breda et al., 2016). Kynurenic acid offers brain protection by blocking the effect of stimulating neurotransmitters at NMDA receptors. Quinolinic acid is neurotoxic and can cause inflammation and cell death in Alzheimer’s and Parkinson’s diseases by activating the NMDA receptors (Lim et al., 2017). Other microbial metabolites also contribute to the functionality of the CNS (Lovelace et al., 2017). SCFAs that are produced by gut bacteria may modulate inflammation within the brain. In the same way, bile acids and LPS, derived from gut microbiota, influence neuroimmune functions and can promote neurodegenerative processes (Guillemin et al., 2007). Such metabolites highlight the role of gut microbiome to influence brain function and illness (Schwarcz et al., 2012). Recent studies identified metabolic imbalance in the kynurenine pathway during the progression of Huntington’s disease that proposes new therapeutic targets (Zhang et al., 2019). In the mentioned discoveries, kynurenine is one of the basic metabolites of tryptophan that further undergoes degradation to synthesize neuroprotective or neurotoxic products. A balance between these neuro-protective or toxic products provides normal function of the brain cells that is to maintain regular activities (Maddison and Giorgini, 2015). Huntington’s, Alzheimer’s, and Parkinson’s diseases have been especially the focus of studies. In these conditions, changes in tryptophan metabolism result in an imbalance in the metabolites of the kynurenine pathway that contributes to disease progression. For example, in Huntington’s disease, the kynurenine pathway is likely to be very significant, given that changed levels of kynurenine and its metabolites might have great relevance with neurodegenerative processes (Maddison and Giorgini, 2015; Giorgini et al., 2008). In addition, the implication of tryptophan products such as 5-HIAA and KYNA appears to be a rather well-investigated issue. These metabolites enhance the activity of a host of enzymes, including neprilysin that is involved in clearance of amyloid-beta peptides, a signature constituents of Alzheimer’s (Maitre et al., 2020). The imbalance in a number of tryptophan metabolites triggers off the appearance of a variety of neurological and psychiatric pathologies. Therefore, this metabolic pathway is of great importance fundamental to brain health and pathology (Roth et al., 2021). Another important area of research concerning gut bacteria relevant to the health of the brain is related to bile acid metabolism. Metabolism of bile acids by gut microbes is a key physiological process; it may reach the brain through key regulators of the gut-liver, gut-brain, and gut-testis axes (Lin and Ma, 2023). This gut bacteria metabolized bile acid seems impaired in Alzheimer’s and, therefore, has a specific role in gut-to-brain communication (MahmoudianDehkordi et al., 2019). The influence of the gut microbiome on bile acid profiles starts to show complex communication between microbial and host metabolisms and thus opens a window for deeper understanding and possible therapeutic intervention in neurodegenerative pathologies.

Lipopolysaccharides are other microbial metabolites may have the ability to induce neuroinflammation in the context of neurodegenerative diseases. Quinovic acid glycosides, provided by the gut microbiota, have been proposed as activators of neuroinflammatory pathways, including those potentially relevant to Parkinson’s disease (Montoro et al., 2004; Grasselli et al., 2017; Di Giorgio et al., 2006). It supports the activation of microglia-resident immune cells in the brain and the release of pro-inflammatory cytokines that, besides their wide-ranging effects, may also induce cytotoxicity toward neuronal cells. Neuroinflammation caused by LPS reduces hippocampus progenitor cell proliferation, survival, and differentiation and, in such a way, links it more closely to neurodegenerative processes (Russo et al., 2011). Astrocyte TLR-4 activation through LPS also causes neuron death along with related neuroinflammation capable of altering neuron membrane cholesterol. Neuroinflammation further coexists with chronic systemic inflammation and BBB damage with chronic systemic inflammation and during LPS induction, which further leads to neurological diseases (Kalyan et al., 2022).

Microbial metabolites reduce neuroinflammation-inflammation believed to be a key factor to develop neurodegenerative conditions. For example, certain SCFAs are known to modify microglial activation in Parkinson’s Disease that indicates a possible route to reduce disease progression (Haase et al., 2020). These metabolites produced by the gut microbiota through various mechanisms adjust the inflammatory processes of the brain. They cross the BBB and modulate the activity of microglial cells-immune cells of the brain that regulates the inflammatory responses in the CNS (Haase et al., 2020). It was evidenced that immunomodulation and demyelination processes, through microbiota-derived metabolites action, open new treatment options (Rebeaud et al., 2022). For example, the oral administration of germ-free mice with a defined subset of microbial metabolites induced neuroinflammation and motor dysfunctions in animal models of Parkinson’s disease. It suggests that such metabolites may affect the brain health (Sampson et al., 2016). The microbial proteins or their metabolites may further contribute to neurodegeneration either by causing amyloid formation by human proteins or by amplifying an inflammatory response against endogenous neuronal amyloids. Again, this suggests a direct relationship between microbial metabolites and the processes underlying neurodegenerative diseases (Friedland and Chapman, 2017). One also finds a two-way interaction between gut microbiota and the CNS in the production of microbial metabolites that support stability in the CNS, thereby reducing the incidence and degree of pathogenic neuroinflammation (Trichka and Zou, 2021).

## Molecular mechanisms and pathways

The complex molecular interaction between the gut microbiota and the brain plays a critical role in the pathogenesis of neurodegenerative diseases (Begum et al., 2022). These interactions are integral to the concept of the microbiota-gut-brain axis, which has increasingly been recognized to play a clinically relevant pathophysiological role in neurodegenerative diseases such as Alzheimer’s and Parkinson’s (Kowalski and Mulak, 2019). Such interactions may finally cause epigenetic changes, including DNA methylation and histone modifications, important in understanding the initiation and use-dependent developing process of neurodegenerative disease (Begum et al., 2022). The gut microbiota shows different pathways regarding the communication with the brain (Ceppa et al., 2020). The enteric nervous system has often been referred to metaphorically as a “second brain,” which plays an intrinsic role in transmitting signals to the brain from the gut; the vagus nerve signals are of considerable importance (Cox and Weiner, 2018). Gut microbiota dysbiosis is associated with the impairment of both BBB and the promotion of neuroinflammation, eventually driving the pathogenesis of Alzheimer’s and other diseases (Kowalski and Mulak, 2019).

This also encompasses neural inflammation, which is a critical player in the pathogenesis of neurodegenerative diseases (Chidambaram et al., 2022). The composition and function of the gut microbiota itself are major drivers for the host’s overall immune response (Padhi et al., 2022). With all this information, dysbiosis could lead to local and systemic immune-mediated inflammation and possibly result in neuroinflammation, which is already well-documented to contribute to neurodegeneration (Mou et al., 2022). This is well evidenced in conditions like Alzheimer’s, where the gut microbiota composition has been associated with disease immunopathogenesis (Leblhuber et al., 2021). Chronic inflammation may also be caused by this dysbiosis of the gut microbiota, which elicits systemic chronic inflammation by means of pro-inflammatory cytokines and bacterial cell wall components that are released. This promotes the entry of neurotoxic substances and inflammatory mediators into the brain and amplifies their neurodegenerative properties (Lukiw, 2020). In some diseases, like multiple sclerosis, the microbial metabolites coming from the gut can translocate across the BBB and further expand their immune cell-mediated effects within the CNS (Dopkins et al., 2018). Further, dysbiosis of the gut microbiome takes part in the change of BBB permeability, participates in the CNS, and takes part in the mechanisms of neurodegenerative diseases (Mou et al., 2022; Rutsch et al., 2020). A better understanding of how gut microbiome dysbiosis contributes to neuroinflammation creates opportunities for therapeutic interventions (Boeri et al., 2021). Targeting the gut microbiome may create opportunities for systemic and neural modulation of inflammation and potentially offer a new approach to treat or slow neurodegenerative diseases (Leblhuber et al., 2021).

The integrity of the gut and BBB is crucial for health, and their dysfunction was underlined as important in the pathophysiology of neurodegenerative diseases (Mou et al., 2022). The gut barrier forms a very important barrier that promotes and maintains health within the gastrointestinal and systemic environments. It acts as a selective barrier, absorbing nutrients beneficial to the body while excluding noxious substances and pathogenic organisms from entry into the general bloodstream (Kowalski and Mulak, 2019). The gut barrier function has been directly connected with the gut microbiota, which contributes to regulating immune responses (Parker et al., 2020). Disturbances of the gut microbiota will lead to increased gut permeability, commonly called “leaky gut,” linked to various disorders (Kelly et al., 2015; Nagpal and Yadav, 2017). The BBB is a selective and protective barrier that separates circulating blood from the brain and protects the brain from injury by pathogens. Gut microbiota disorders have been associated with destruction of the BBB. Therefore, a healthy gut microbiota is crucial to defend the BBB (Tang et al., 2020). Proper maintenance of this barrier is supported by the gut microbiome, which is closely linked to the BBB and active neuroinflammation — key elements in neurodegenerative disease (Braniste et al., 2014). Optimal health of the gut and BBB is essential to maintain overall health and is significantly influenced by the gut microbiome. The alteration in gut microbiota composition leads to leaky gut barrier and disturbed functions of BBB contribute to the onset of neurodegenerative disease development (Kowalski and Mulak, 2019; Mou et al., 2022). Such interactions provide ways for therapeutic opportunities by targeting the gut microbiome to maintain barrier integrity and reduce neurodegenerative processes (Riccio and Rossano, 2019).

Genetic predisposition greatly affects the gut microbiome and its interaction with the brain (Ning et al., 2022). For example, changes in gut microbiota at the class levels of Blautia and Gammaproteobacteria have been linked to neuropsychiatric conditions such as Alzheimer’s disease, major depressive disorder, and schizophrenia (Zhuang et al., 2020). These suggest that genetic susceptibility may contribute to an increased risk of neurodegenerative diseases due to altered compositions in the microbiome (Martínez Leo and Segura Campos, 2020). It has been suggested that dietary patterns high in fiber and low in saturated fat shape a healthier host’s gut microbiome. This may possibly even gradually protect against neurodegeneration (Gubert et al., 2020). The interplay of both genetic and environmental factors reveals therapeutic targets within the gut-brain axis for neurodegenerative diseases that are revealed (Fang et al., 2020). Additionally, modulation of the gut microbiome is being considered for new preventive and therapeutic approaches. These approaches include diet, probiotics, or other interventions (Kowalski and Mulak, 2019). These results are obtained by further in-depth research into the complex interaction between genetics, gut microbiome, and the environment. They might in the near future enable individual microbiome profiling. This profiling could support the design of tailored personalized strategies for prevention and treatment of neurological diseases (Wang H. et al., 2021).

A challenge in understanding the mechanisms that link gut microbiome to neurodegeneration is the complex nature of the interactions (Solanki et al., 2023). The gut microbiome influences the brain via several direct modes that include neuronal communication, endocrine mediators and immune signaling (Martin et al., 2018). It is difficult to narrow down and point to particular causal relationships and mechanisms with such complexity (Padhi et al., 2022). It is difficult to narrow down and point to particular causal relationships and mechanisms with such complexity (Cryan et al., 2019; Westfall et al., 2017). Changes in the gut microbiota can disrupt the BBB, reduce neuroinflammation and lead to neural injury and neurodegeneration (Kowalski and Mulak, 2019). However, the mechanisms behind these effects are uncertain. It is clear that both new technologies and better research tools should integrate fully to solve the causative role of gut microbes in neurodegeneration (Raimondi et al., 2020). Further investigations are needed to understand how changes in gut microbiota can promote neurological resilience (Raval et al., 2020). Only when these unknown complex molecular mechanisms linking gut microbes to health are understood, new treatments are likely to be successfully developed (de Vos et al., 2022).

## Therapeutic potential of microbiome

Reports have shown changes in the gut microbiota associated with neurodegenerative disorders. These include Parkinson’s disease, Alzheimer’s disease, multiple sclerosis, and autism spectrum disorders (Bashir and Khan, 2022). Although the molecular mechanisms responsible for this activity have provided a basis for understanding, much remains to be explored regarding the modulation of neuroinflammation and neurodegenerative processes through gut-derived bacterial strains (Ahmed et al., 2019). In addition, prebiotics and probiotics influence the gut microbiome through rebalancing the gut microbiome. It can also decrease the risk of neurodegenerative and cerebrovascular diseases (Koszewicz et al., 2021).

Several preclinical studies and clinical trials deal with its therapeutic potential against neurodegenerative diseases by modulation of gut microbiota (Peterson, 2020). Examples are the use of FMT derived from healthy donors, which improved motor function and reduced neuroinflammation in preclinical studies using mouse models with induced Parkinson’s disease (Sampson et al., 2016), while ongoing clinical trials conducted to see the effects of probiotic supplementation in Alzheimer’s patients yielded possible benefits regarding cognitive function and lowering inflammatory markers (Naomi et al., 2021). Each of these illustrates the growing interest in microbiome-targeted therapeutics to treat neurodegenerative diseases. Moreover, environment-based factors, including physical exercise, diet, and stress, trigger or alter the gut microbiome and thus may become novel therapeutic strategies in neurodegenerative diseases (Gubert et al., 2020). The gut microbiome holds promise to reduce the pathological burden of neurodegenerative diseases through novel diagnostic and therapeutic strategies (Padhi et al., 2022).

Probiotics are live microorganisms that provide a health benefit to the host when administered in adequate amounts (Plaza-Díaz et al., 2018). They have been considered modulators of the gut microbiome to induce diversity and functionality (Floch and Hong-Curtiss, 2001). Prebiotics are a non-digestive form of food that promotes the growth and activities of beneficial microorganisms residing in the intestines. They act like ‘food’ for probiotics; hence, consumption of these types maintains healthy gut microbiome (Gibson and Roberfroid, 1995). Probiotics and prebiotics change the gut microbiota composition with an improvement in cognitive scores and possibly delay the disease pathology of Alzheimer’s disease (Kesika et al., 2021). Probiotics and prebiotics also decrease neuroinflammation and modulate the barrier function and neurotransmitter activity and, thus, may be used as therapeutic strategies in the management of Parkinson’s disease (Wang H. et al., 2021). The development of effective probiotic and prebiotic therapies is complicated by several compounding factors. The efficacy of the applied strains in modulating the gut microbiome for desired health outcomes depends on their specificity. Dosage and individual variability are also critical determinants. This is because interindividual variations in the composition of human gut microbiota make it impossible for a specific probiotic or prebiotic to have the same effect in different individuals (Lorente-Picón and Laguna, 2021).

Diet has been considered one of the most essential determinant factors in the composition and function of gut microbiotas. Prebiotic-like stimulation of benevolent bacteria by plant-based diet components, including fiber, polyphenols, and unsaturated lipids, is therefore facilitated. In contrast, a diet rich in saturated fats and high in processed foods can lead to dysbiosis or an imbalance of the normal gut microbiome (Polito et al., 2020). The Mediterranean diet is characterized by high consumption of fruits, vegetables, whole grains, and healthy fats. This diet is linked to a healthier gut microbiome and is associated with a lower risk of cognitive decline. Diets high in fiber can promote high diversity and health of the gut microbiome, which might further provide benefits for neurodegenerative diseases (Kang and Zivkovic, 2021). Evidence supports dietary intervention in modulation of the gut microbiome and its interactions with neurodegeneration. Dietary supplementation with probiotics has improved cognitive function, especially in subjects suffering from mild cognitive impairment as a precursor to Alzheimer’s disease (Zhu et al., 2021). Further, prebiotics are potential enhancers of gut microbiota composition and improve mental health outcomes in Alzheimer’s Disease (Kang and Zivkovic, 2021).

The potential exists for gut microbiome remodeling and replenishment to reduce risk from neurodegenerative and cerebrovascular diseases. The lumination into new drugs and compounds against the gut microbiome is rapidly ongoing. These include prebiotics, medicinal herbs, probiotics, and synbiotics that come under this umbrella. They may have potential therapeutic modulations for the microbiota-gut-brain axis in neurogenerative diseases such as Parkinson’s and Alzheimer’s. It is under investigation that personalized diet and oral bacteriotherapy may postpone Alzheimer’s disease through modification of neuronal pathways or ways of information processing (Goyal et al., 2021; Peterson, 2020). Personalized approaches will shape the future of microbiome-based therapies. This includes systems biology-designed personalized anti-inflammatory diets. It also involves multi-functional drug design targeting multiple CNS areas. These approaches aim to provide symptomatic efficacy and neuroprotective properties against neurodegenerative disorders (Rosario et al., 2020; Youdim and Buccafusco, 2005).

Safety, ethical and regulatory questions arise when applying microbiome research clinically. It is unclear what role and possible mechanisms of the gut microbiome in altering vulnerability to neurodegenerative diseases necessitate further human studies to develop effective treatment strategies (Fang et al., 2020). Only then do individual variations in microbiome composition affect therapeutic strategies based on the microbiome and hence individualized approaches are required for each clinical application. Implementation to clinical practice requires identification of key taxa and functional microbial pathways driving host physiology. Such therapies are thus translated through collaborative networks of medical centers and research institutes to bring precision medicine to neurodegenerative diseases from bench to bedside (Strafella et al., 2018).

## Biomarkers and diagnostic advances

Microbiome-based biomarkers not only allow early detection but also disease progression monitoring and personalized medicine approaches. Altered gut microbiome instability (probably during the beginning or progression of neurodegenerative diseases) may be characterized by microbial signatures imposed by the abnormal nature. They could be utilized as potent and non-invasive diagnostic means to help monitor patients who suffer from neurodegenerative disorders (Török et al., 2020). On the other hand, the CNS influences the status of the microbiome and vice versa: while the gut microbiome in itself is a key function in modulating neurodegenerative diseases (Ghezzi et al., 2022). Such bidirectional communication between the gut and brain may be disrupted by neurodegenerative conditions (Chidambaram et al., 2022). A similar mechanism could explain disturbances in gut motility and thus in the gut environment leading to change in microbiome composition (Mulak and Bonaz, 2015), such as autonomic nervous system dysfunction which is prominent in Parkinson’s disease. Alzheimer’s disease appears to be characterized by increased gut permeability and microbiota diversity altered through neuroinflammatory and dysregulation of the stress hormones (Kesika et al., 2021). This complex interaction between the CNS and gut microbiome may explain how alterations within the brain influence gut microbiota (Rueda-Ruzafa et al., 2020). More recent studies have found that there are actually identifiable biomarkers in the microbiome for neurodegenerative diseases. For example, changes in the gut microbiota are associated with clinical features and may serve not only as a marker for disease progression but also in response to treatment (Wang Q. et al., 2021). Higher risk of amyotrophic lateral sclerosis (ALS) has been associated with other genera. However, levels of glutamine and other metabolites generated in the gut microbiota are negatively related to Alzheimer’s and Parkinson’s diseases (Ning et al., 2022). These findings support the use of detectable early microbiome changes to predict neurodegenerative disease onset or progression.

Recent advances in microbiome profiling technologies allow us to investigate complex microbial communities in the human body. Next-generation sequencing technology has revolutionized microbiome research through metagenomics and metatranscriptomics and allowed high-throughput analyses of microbial communities at exceptional depths. Metabolomics that analyzes metabolites within biological systems has clarified how microbial metabolites influence host physiology and disease states (Holmes et al., 2011). The coupling of technologies, computational tools and bioinformatics, in particular, directly brought about enhanced accuracy and sensitivity to the analysis of microbiomes and their possibility as a diagnostic tool. These improvements have helped us better understand the impact of the microbiome to health and disease and opened up new avenues to develop microbiome-based diagnostics and therapeutics (Jiang et al., 2020; Schlaberg, 2020).

Standardizing and validating microbiome analysis remain major challenges in developing microbiome biomarkers for clinical application. The complexity of the microbiome itself demands more homogeneous high-throughput analyzers and a framework in which confidentiality for information is maintained yet integrated with other tests in laboratories (Pritzker and Azad, 2004). Furthermore, a technique like enzyme-linked immunosorbent assay (ELISA) is both systemically and randomly variable in quality, adding more errors and hence requiring tighter control over the performance of assays for use in clinical settings (Andreasson et al., 2015). Developing and validating host serologic microbial biomarkers in diseases, particularly inflammatory bowel diseases, have reached a unique place in diagnosis and prognosis but still present difficulties in being standardized and interpreted (Dubinsky and Braun, 2015). Another challenge is the interpretation of generated complex data obtained through studies on the microbiome. The complexity of microbial communities and their interactions with the host makes it challenging to derive clinically relevant insights from microbiome data. Microbiome study’s amplicon-based marker gene approaches are subject to error or bias because of several steps of following generation library sequencing preparation (Gohl et al., 2016). Of importance also is the fact that a robust and standardized procedure for sample preparations should immunize the microbiome markers from research into clinical application (Freiwald and Sauer, 2009).

New research areas involve gut microbiome applications in diagnostics using novel biomarkers. For example, the BactoChip microarray is a culture-independent technique to identify bacterial species and their relative abundance within complex communities. It could help improve clinical detection and discrimination of bacteria (Ballarini et al., 2013). The potential for integrating microbiome markers with other biological markers, such as genetic and proteomic markers, is being studied to further the diagnostic accuracy and monitoring efficiency achieved with disease methods. Combination of logistic regression and Linear Discriminant Analysis, which is a statistical method used to classify data patterns, potentially performs better than other methods in identifying relevant markers from high-dimensional mass spectrometry data during cancer diagnosis. Other assays and technologies, like gene expression profiling, MALDI-TOF MS, which is an advanced technique used to identify bacteria and other microorganisms, and nucleic acid aptamers, which are special molecules that can bind tightly to specific targets, such as proteins, are continually reported to have potential use for infectious disease diagnosis and management (Mitsuma et al., 2013).

## Conclusion

This review discussed how the gut microbiome contributes to neurodegenerative diseases through the gut-brain axis. The gut microbiome plays a key role in brain health. Probiotics, prebiotics, dietary changes, and FMT show promise in treating neurodegenerative diseases like Alzheimer’s and Parkinson’s. However, turning microbiome therapies into effective treatments is challenging because of the need for standardized analysis methods and personalized approaches. Future research should focus on understanding the specific mechanisms of gut-brain communication using multi-omics data. Long-term studies are needed to connect microbiome changes with the progression of neurodegenerative diseases. Collaborative research across fields can turn microbiome insights into new, effective treatments that improve patient outcomes.
